# Cerebellar theta burst stimulation does not improve freezing of gait in patients with Parkinson’s disease

**DOI:** 10.1007/s00415-017-8479-y

**Published:** 2017-04-05

**Authors:** Arno M. Janssen, Moniek A. M. Munneke, Jorik Nonnekes, Thomas van der Kraan, Alice Nieuwboer, Ivan Toni, Anke H. Snijders, Bastiaan R. Bloem, Dick F. Stegeman

**Affiliations:** 1 Department of Neurology, Donders Institute for Brain, Cognition and Behaviour, Radboud University Medical Center, Nijmegen, The Netherlands; 2 Department of Otorhinolaryngology, Donders Institute for Brain, Cognition and Behaviour, Radboud University Medical Center, Nijmegen, The Netherlands; 3 Department of Rehabilitation, Donders Institute for Brain, Cognition and Behaviour, Radboud University Medical Center, Nijmegen, The Netherlands; 4 Department of Rehabilitation Sciences, KU Leuven, Louvain, Belgium; 5 Centre for Cognitive Neuroimaging, Donders Institute for Brain, Cognition and Behaviour, Radboud University, Nijmegen, The Netherlands; 6 Department of Neurology, Maasziekenhuis Pantein, Boxmeer, The Netherlands

**Keywords:** Parkinson’s disease, Freezing of gait, Freezing of upper limbs, Theta burst stimulation, Cerebellum

## Abstract

Freezing of gait (FOG) in Parkinson’s disease (PD) likely results from dysfunction within a complex neural gait circuitry involving multiple brain regions. Herein, cerebellar activity is increased in patients compared to healthy subjects. This cerebellar involvement has been proposed to be compensatory. We hypothesized that patients with FOG would have a reduced ability to recruit the cerebellum to compensate for dysfunction in other brain areas. In this study cerebellar activity was modified unilaterally by either excitatory or inhibitory theta burst stimulation (TBS), applied during two separate sessions. The ipsilateral cerebellar hemisphere, corresponding to the body side most affected by PD, was stimulated. Seventeen patients with PD showing ‘off’ state FOG participated. The presence of FOG was verified objectively upon inclusion. We monitored gait and bimanual rhythmic upper limb movements before and directly after TBS. Gait was evaluated with a FOG-provoking protocol, including rapid 360° turns and a 10-m walking test with small fast steps. Upper limb movement performance was evaluated with a repetitive finger flexion–extension task. TBS did not affect the amount of freezing during walking or finger tapping. However, TBS did increase gait speed when walking with small steps, and decreased gait speed when walking as fast as possible with a normal step size. The changes in gait speed were not accompanied by changes in corticospinal excitability of M1. Unilateral cerebellar TBS did not improve FOG. However, changes in gait speed were found which suggests a role of the cerebellum in PD.

**Electronic supplementary material:**

The online version of this article (doi:10.1007/s00415-017-8479-y) contains supplementary material, which is available to authorized users.

## Introduction

Freezing of gait (FOG) is a disabling feature of Parkinson’s disease (PD), resulting in mobility problems and frequent falls [1, 2]. FOG is an episodic phenomenon, characterized by brief periods of inability to step effectively [3]. FOG is not present in all patients, but becomes more common in advanced PD [4]. The mechanism behind its occurrence is still not clear.

We focus on the possible role of the cerebellum in PD, and specifically on its role in the pathophysiology underlying FOG. Although lesions in a single brain area can occasionally induce FOG [5], FOG generally results from a widespread dysfunction within a neural gait circuitry involving the supplementary motor area (SMA) [6], the mesencephalic locomotor region [6, 7] and the cerebellar locomotor region [8, 9]. Recent work has emphasized the tight interplay between the cerebellum and the basal ganglia [10, 11]. Cerebellar activity is increased in PD patients compared to healthy subjects [12, 13]. This hyper-activation in the cerebellum may be an adaptive mechanism that compensates for the defective basal ganglia [11, 12, 14–16].

We hypothesize that compared to patients without FOG, patients with FOG are less able to recruit the cerebellum to compensate for dysfunction of other brain circuitries. This hypothesis is supported by the fact that FOG is common in patients with progressive supranuclear palsy (PSP) [17], who also have lesions in brainstem areas connected with the cerebellum [18].

To investigate the possible compensatory role of the cerebellum in PD patients with FOG, we intended to up-regulate cerebellar activity with transcranial magnetic stimulation (TMS) [19, 20], and to measure the resulting effect on freezing and movement performance using a specific set of tasks. The tasks included a FOG-provoking gait protocol, including rapid 360° turns [21] and a 10-m walking test with small fast steps [22, 23], as well as a repetitive finger flexion–extension task, which can evoke upper limb freezing (FOUL) [24, 25]. The severity of FOUL correlates with FOG scores, but not with disease severity, which supports the hypothesis that a generic motor control problem partially underlies freezing in both the upper and the lower extremities [26].

Theta burst stimulation (TBS), a specific type of repetitive TMS (rTMS) [20], is a suitable stimulation protocol for patient studies. It combines a short stimulation period (40–190 s) with long lasting (up to 1 h) effects on cortical excitability that are either inhibitory or excitatory [27]. Following the rationale of previous studies [28–30], we used an excitatory TBS form (intermittent TBS, iTBS) to stimulate the cerebellum and hypothesized that this should improve both general gait and upper limb performance, including a reduction in freezing episodes. As a control condition, we stimulated the cerebellum with a protocol that aimed to achieve the opposite effect, i.e. inhibitory continuous TBS (cTBS). If the increased cerebellar activity is indeed compensatory, this control condition should either worsen or (in case of ceiling effects) not affect freezing episodes in the upper and lower limbs.

## Methods

### Subjects

Fifteen patients (12 men) were included in all analyses and two additional patients (one male) were included only in analyses of gait, pegboard test and corticospinal excitability. Clinical and demographic characteristics of all 17 patients are listed in Table 1. Three additional patients could not be included in any analyses. Two dropped out due to uncomfortable co-activation of neck muscles during TBS, one because the protocol was experienced as too stressful.

**Table 1:** Clinical and demographic characteristics of 17 Parkinson’s disease patients

Parameter	Mean	Range
Age (years)	61.2	46–76
Parkinson’s disease duration (years)	8.5	1–25
FOG duration (years)	3.4	1–12
Hoehn and Yahr stage		2–3
MDS-UPDRS part 3	33.4	12–68
NFOGQ	16.5	3–28
FAB	16.0	12–18
MMSE	28.5	24–30
Resting motor threshold (%MSO)	43	34–60

For MDS-UPDRS, N-FOGQ and Hoenh and Yahr stage, higher scores indicate worse functioning. For both FAB and MMSE, lower scores indicate worse functioning. The scores were evaluated ‘off’ medication

*MDS-UPDRS* Movement Disorder Society—unified Parkinson’s disease rating scale part 3 (score 0–132), *N-FOGQ* new freezing of gait questionnaire (score 0–28), Hoenh and Yahr stage (score 0–5), *MMSE* mini mental examination (score 0–30), *FAB* Frontal Assessment Battery (score 0–18)

Patients had objectively verified FOG. FOG was objectified by expert raters using standardized and established FOG-provoking methods [21, 23]. Exclusion criteria were neurological disorders other than PD, presence of deep brain stimulation, a Mini Mental State Examination (MMSE) [31] score <24, and exclusion criteria for TMS experiments [32]. All subjects gave written informed consent prior to participation. The ethics committee of the Radboud University Medical Centre approved the study, which was performed in accordance with the Declaration of Helsinki.

### Experimental design

**Fig. 1:**
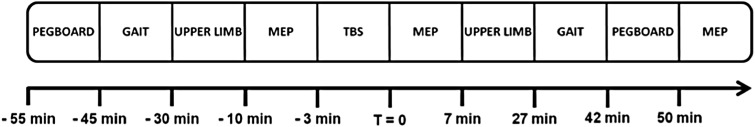
Protocol-design for a session. All post-TBS measurements were performed in 30–60 min, depending on the patients’ performance. The added timeline is a rough indication (in minutes, and the moment directly after the TBS set to 0). Not included are N-FOGQ, MMSE, FAB and MDS-UPDRS part 3, for which the scores were determined prior to this protocol in session one

Testing occurred while patients were in a practically defined ‘off’ state; i.e. after withholding all anti-parkinsonian medications for at least 12 h. To create a homogenous patient group, we included only patients with ‘off’ state FOG, as this is the most common type of FOG [33, 34]. Prior to testing, clinical data were collected including the new freezing of gait questionnaire (N-FOGQ) [35], MMSE [31], frontal assessment battery (FAB) [36] and the Movement Disorder Society Unified Parkinson’s disease rating scale (MDS-UPDRS) part 3 [37]. Patients were stimulated with cerebellar iTBS and cTBS in separate sessions. During the first session, patients were stimulated with cTBS or iTBS; during the second session they received the opposite TBS protocol, always in a counterbalanced manner. Patients were kept unaware of the nature of the stimulation and the nature of the expected effects. The researcher was aware, as he was involved in both the clinical testing as well as the stimulation protocol. The sessions were at least one week apart to ensure sufficient wash-out of the preceding TBS. Before and after TBS, patients performed a gait protocol and rhythmic upper limb task to measure the effect on movement performance and freezing duration (Fig. 1). In addition to these primary outcome measures, cortical excitability was measured with motor evoked potentials (MEPs), and patients performed a pegboard dexterity task to objectively quantify upper limb bradykinesia [38, 39].

### Theta burst stimulation

TBS [27] was administered using a C-B60 figure-of-eight coil (MagVenture, A/S, Farum, Denmark), connected to a MagPro X100 (MagVenture) stimulator. The ipsilateral cerebellum (1 cm below and 3 cm lateral to inion) was stimulated, corresponding to the most affected side by PD based on the MDS-UPDRS part 3 (i.e. the body side with the highest scores). The coil was placed tangentially to the scalp with the handle pointing upwards. To ensure anatomically identical coil positioning during and over sessions, location and orientation of the coil target position were saved using a stereotactic image guidance system (Localite TMS Navigator, Localite GmbH, Sankt Augustin, Germany). Cerebellar TBS was administered with an intensity of 70% of resting motor threshold (see “Corticospinal excitability” in section “Methods”). The stimulation period for cTBS was 40 s and for iTBS 192 s.

### Gait protocol

Occurrence of FOG was measured using a protocol that is known to elicit FOG. This protocol included eight 360° turns (as fast as possible, four times clockwise, four times counterclockwise) [21] and a 10-m gait trajectory (including gait initiation and gait termination while reaching a destination (stripes on the floor)), using different velocities (self-selected speed = normal; and as fast as possible) and different stride lengths (self-selected stride length = normal steps; and 20% of leg-length = small steps) [22, 23]. Visual guidance for the small steps was provided with stripes on the floor for three steps at the beginning and at the end of the gait trajectory.

The entire gait protocol was videotaped allowing for offline assessment of FOG. Two independent, experienced, and fully blinded raters scored the videos for the presence and duration of FOG. The definition used to score FOG was an obvious episode with ineffective stepping and the characteristic FOG phenotype. When raters disagreed, trials were sent back for consensus. FOG seen when turning after the 10-m gait trajectory was not included in the analysis.

The time to complete each task (execution time) was determined to measure general gait performance. A decrease in execution time may be due to increased gait speed as walking is easier and less likely to be driven to the threshold for FOG [40]. Therefore, a decreased execution time was interpreted as increased gait speed and as improved performance.

### Upper limb task

To elicit FOUL, the instruction was to make anti-phase rhythmic flexion and extension movements using both index fingers as described previously [24, 25, 41]. Two different amplitudes [45° (normal) or 30° (small)] and two different movement frequencies [normal (100%) or fast (133%)] were used. “Normal frequency” was defined as the patients’ specific comfortable movement speed, determined for each subject individually at the beginning of the first session. The four different conditions were: normal amplitude + normal speed (NANS), normal amplitude + fast speed (NAFS), small amplitude + normal speed (SANS), and small amplitude + fast speed (SAFS). SAFS has proven to be the most sensitive condition to elicit FOUL [25]. Each condition was repeated three times, both pre- and post-TBS. Auditory pacing guided the first six movement cycles. After auditory pacing stopped, the patients had to maintain the rhythm for 25 s. Both hands were covered to prevent visual feedback. Angular finger displacement was registered with single axis goniometers (Type F35, Biometrics Ltd., Newport, UK) placed over the metacarpophalangeal joint of the index fingers.

The data of the goniometers were processed and analysed with Matlab (MathWorks, Natick, Massachusetts, USA). For each condition the peak-to-peak amplitude and frequency values were calculated per movement cycle.

For each pre- and post-measurement the mean duration of freezing during a complete trial was defined. In accordance with Vercruysse and co-workers [25], the beginning of a freezing episode was determined as “the onset of abnormally small motion cycles (<50% of the initial amplitude) accompanied by an irregular cycle frequency”, which proved to be a reliable procedure. The end was defined as the moment where movement cycles with regular amplitude and frequency were resumed, or when the trial ended. A semi-automatic detection was used, which was visually checked and corrected by two independent raters.

### Pegboard dexterity test

The pegboard dexterity test [38, 39] was used to determine upper limb bradykinesia at the start and end of each session as a brief surrogate test to estimate overall treatment effects and disease state. This test strongly correlated with the overall MDS-UDPRS part 3 score [38, 39] and repeating the entire MDS-UPDRS part 3 was considered to be too cumbersome for patients. The time needed to turn four wooden pegs upside down using one hand, from one hole into the next, was recorded four times for each hand. The average over the four trials was taken for each hand separately.

### Corticospinal excitability

With single pulse TMS corticospinal excitability of the primary motor cortex (M1) was determined. The pulses were administered using the figure-of-eight coil. The optimal location of the coil for eliciting MEPs in the resting first dorsal interosseous (FDI) muscle of the most affected hand was tracked (hotspot). To ensure identical coil positioning during and over sessions, the location and orientation of the coil over the hotspot were also saved using the stereotactic image guidance system. The resting motor threshold was determined, defined as the minimum stimulator intensity required to obtain MEPs with an amplitude of at least 50 μV in at least five out of ten trails in the relaxed FDI of the most affected hand. Last, the minimum stimulator intensity was determined to obtain single pulse MEPs of on average 1 mV over 10 trials (SI_1mV_). Directly before (pre) TBS, directly after TBS (post 1), and at the end of the session (post 2), 20 single pulses at SI_1mV_ were applied to measure the corticospinal excitability.

### Statistical analyses

All statistical analyses were performed in IBM SPSS Statistics 20. The data for the upper limb task, the gait protocol, and the pegboard dexterity test were all separately analysed using the ANOVA with random factor ‘patient’ and fixed factors ‘stimulation’ (cTBS or iTBS) and ‘time’ (pre or post). The fixed factor ‘task’ was added for the analyses of the upper limb task (NANS, NAFS, SANS or SAFS) and for the gait protocol (normal, fast, small steps or small fast steps). The analyses for the upper limb task and the pegboard dexterity test were performed separately for the most and least affected hand as any difference between hands was not part of the research question. Additional ANOVAs for both stimulation protocols (cTBS and iTBS) with random factor ‘patient’ and fixed factor ‘time’ (pre or post) were performed to explore the effects of excitatory or inhibitory stimulation separately.

The main variables of interest were the mean FOG duration (per trial) in the gait protocol and the mean FOUL duration (per trial) in the upper limb task [42]. In addition to freezing duration, the mean execution time in the gait protocol, and the mean peak-to-peak amplitude and mean frequency in the upper limb task (calculated over the complete trials) were evaluated. The variable for the pegboard dexterity test was execution time. In case the fixed factors ‘stimulation’, ‘time’ or an interaction between factors was statistically significant regarding the tested variables post hoc analyses were performed using paired sample *t* tests.

A change in corticospinal excitability was tested comparing the MEP amplitudes of all three time points (pre, post 1, post 2) using a repeated measurements test and for two time points (pre and post 1) a paired sample *t* test. These comparisons for MEP amplitudes were done for cTBS and iTBS separately.

For all analyses, a *p* value <0.05 was considered significant for the ANOVAs. For the post hoc analysis a Bonferroni correction was applied and a *p* value of 0.05/(number of comparisons) was considered significant. All data are shown as mean ± standard error of mean (SEM).

## Results

### Gait: FOG

The gait protocol successfully provoked FOG in 12 out of 17 patients (71%). The other five patients did not show any FOG during the experiments, although they had showed unequivocal FOG episodes during earlier assessments to decide about inclusion in the study. Among the 12 patients who did show FOG, five showed only one or two episodes during the baseline gait measurements. The FOG duration per trial varied from less than 1 s in some patients to a maximum of 357.5 in one patient.

**Fig. 2:**
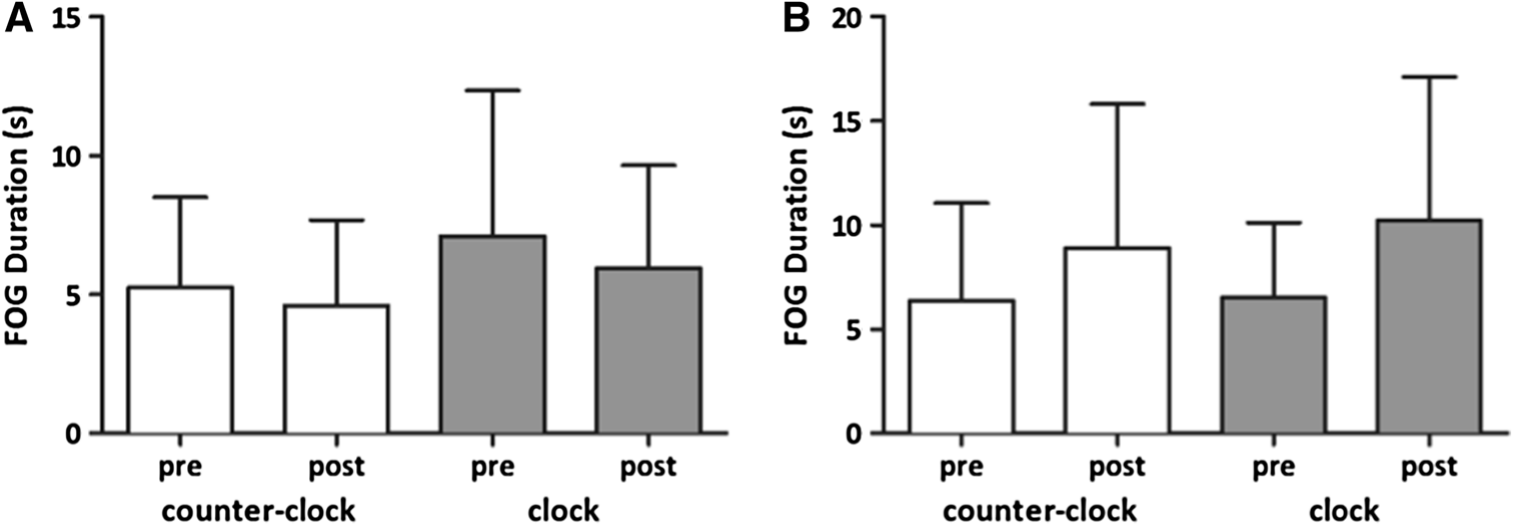
The mean freezing (FOG) duration during turning, before and after stimulation, for the **a** excitatory iTBS and **b** inhibitory cTBS in seconds. The *error bars* signify the SEM

No significant effect of stimulation (cTBS versus iTBS), time (pre versus post), task or interaction between factors was found for FOG duration when all turn and gait conditions were included as separate tasks, nor when all turns were combined and all gait trajectory conditions (normal, fast, small steps, small fast steps) were combined (Table 2). Neither did the separate ANOVAs for both stimulation protocols show significant effects (Tables S1 and S2 in the supplementary material). Because of the lack of a significant effect, only the FOG duration results for the turns are shown (Fig. 2), as this is the most FOG-provoking task [21, 23]. No post hoc analyses were performed for FOG duration.

**Table 2:** Statistics gait task and upper limb task

	Time	Stimulation	Task	Stimulation × time	Stimulation × task	Time × task	Stimulation × time × task
FOG duration [combined]	[0.867; n.s.]	[0.874; n.s.]	[0.865; n.s.]	[1.032; 0.326]	[0.971; n.s.]	[0.735; n.s.]	[0.152; n.s.]
FOG duration [separate]	[0.840; n.s.]	[0.901; n.s.]	[1.078; 0.379]	[0.974; n.s.]	[1.074; 0.382]	[1.180; 0.326]	[0.997; n.s.]
Mean execution time [separate]	[4.005; 0.062]	[1.142; 0.301]	**[10.058; 0.000]**	[1.903; 0.186]	[1.045; 0.398]	**[3.214; 0.011]**	[0.826; n.s.]
FOUL duration [most]	[3.218; 0.073]	[3.287; 0.070]	**[19.158; 0.000]**	[0.671; n.s.]	[0.442; n.s.]	[0.565; n.s]	[0.017; n.s.]
FOUL duration [least]	[0.348; n.s.]	[3.801; 0.052]	**[5.883; 0.001]**	[0.697; n.s.]	[1.162; 0.323]	[0.860; n.s.]	[0.657; n.s.]
Amplitude [most]	[0.009; n.s.]	[1.760; 0.185]	**[60.733; 0.000]**	[0.942; n.s.]	[0.302; n.s.]	[0.699; n.s.]	[0.250; n.s.]
Amplitude [least]	[0.661; n.s.]	[1.271; 0.260]	**[87.445; 0.000]**	[0.139; n.s.]	[0.354; n.s.]	[0.437; n.s.]	[0.005; n.s.]
Frequency [most]	[1.772; 0.184]	[2.472; 0.116]	**[11.317; 0.000]**	[2.746; 0.098]	[0.033; n.s.]	[0.285; n.s.]	[0.191; n.s.]
Frequency [least]	[0.709; n.s.]	[0.052; n.s.]	**[17.049; 0.000]**	[0.591; n.s.]	[0.139; n.s.]	[0.381; n.s.]	[0.043; n.s.]

The factors are ‘time’ (pre or post), ‘stimulation’ (cTBS or iTBS) and task. For gait the task includes (normal, fast, small steps, small fast steps, turning clockwise or turning counterclockwise) and for upper limb (NANS, NAFS, SANS or SAFS). Factor task and interactions with factor task in separate FOG duration and mean execution time = [*F*_5,16_; *p*]. All other factors and interactions in FOG and mean execution time = [*F*_1,16_; *p*]. FOG duration was analysed for all gait tasks [separately, 6 task conditions] and for turns and gait trajectory [combined, 2 task conditions] combined. Factor task and interactions with factor task for FOUL duration, amplitude and frequency = [*F*_3,14;_ *p*]. All other factors and interactions for FOUL duration, amplitude and frequency = [*F*_1,14;_ *p*]. Significant results are indicated in bold

### Gait: speed

When comparing the gait speed for all turns and gait conditions separately, a significant main effect of task was found, but not for stimulation or time. The interaction of factors time and task also showed a significant effect (Table 2). There was no difference in performance during baseline measurements in the cTBS and iTBS conditions.

**Fig. 3:**
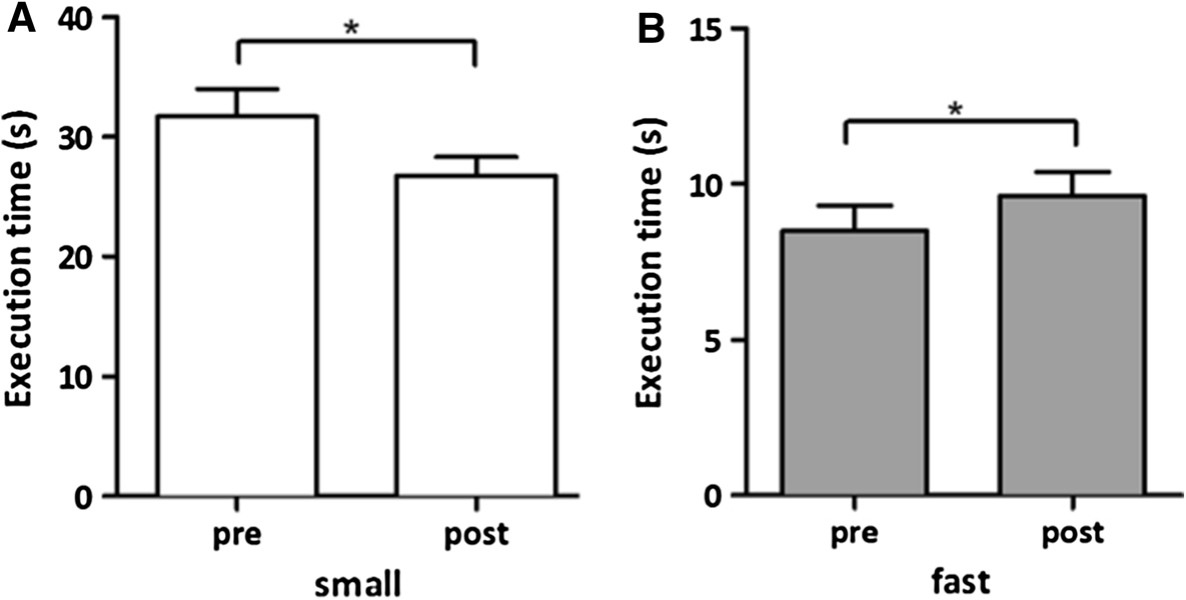
The mean task execution times for the gait protocol (only shown for significant differences) before and after stimulation for the **a** small steps and **b** fast walking normal step size condition in seconds. The *error bars* signify the SEM. The *asterisks* indicate a significant difference between pre- and post-measurements

**Fig. 4:**
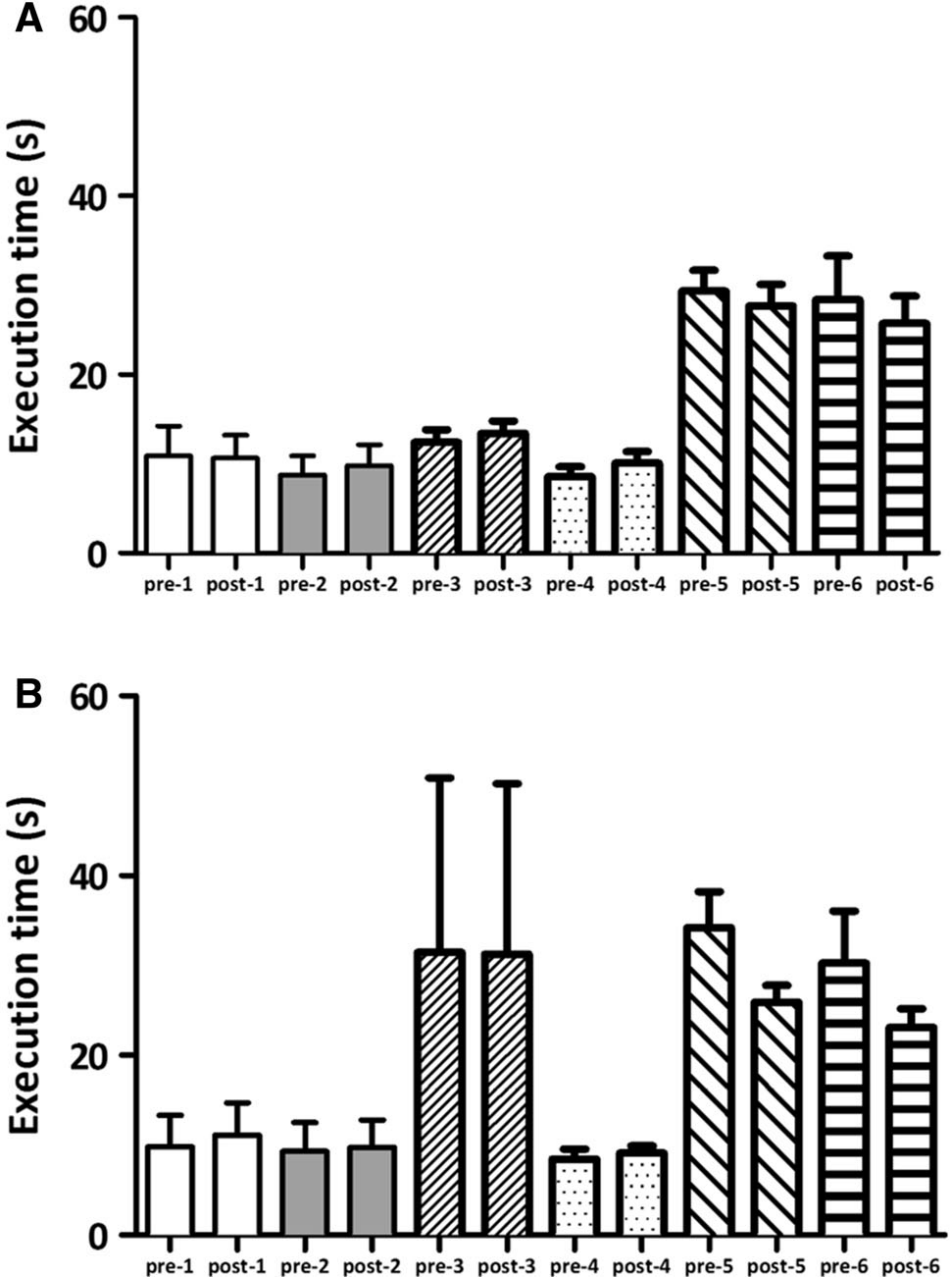
The mean task execution times for the gait protocol before (pre) and after (post) stimulation for the **a** cTBS and **b** iTBS stimulation condition in seconds. The error bars signify the SEM. (pre/post-1 = clockwise turning, pre/post-2 = counter-clockwise turning, pre/post-3 = self-selected speed and normal step size, pre/post-4 = fast walking with normal step size, pre/post-5 = self-selected speed and small step size, pre/post-6 = fast walking with small step size)

Post hoc analyses showed a significant decrease in execution time between pre and post for TBS intervention in the small steps with normal speed condition (Fig. 3a, 32.1 s pre-TBS versus 26.8 s post-TBS; *p* = 0.004). A small, but also significant increase in execution time between pre- and post-TBS in fast walking with normal step size (8.5 versus 9.6 s; *p* < 0.001) was found (Fig. 3b). All other combinations of gait conditions did not show significant effects. The execution times for the gait tasks are shown in Fig. 4.

The ANOVA for the iTBS protocol showed a significant main effect of time and task, as well as for the interaction of factors (Table S1). Post hoc analyses showed a significant decrease in execution time between pre and post for iTBS intervention in the small steps with normal speed condition (34.2 s pre-TBS versus 25.9 s post-TBS; *p* = 0.014). The ANOVA for the cTBS protocol showed only a significant main effect of task (Table S2), which was no reason for further post hoc analyses.

### Upper limb

The upper limb task successfully provoked FOUL at least once in all patients at baseline. The FOUL duration varied strongly from 0.2 to 37.9 s and was 3.1 s on average. In 42% it was shorter than 1 s and in 66% shorter than 2 s. The average duration is shorter than in previous reports [25, 41, 43]. In total 271 trials showed freezing during baseline (both sessions combined), with 54% bilateral, 31% unilateral most affected and 15% unilateral least affected side.

The main factors time and stimulation showed no significant effect on FOUL, nor did any of the interactions between factors (Table 2). The factor task (NANS, NAFS, SANS or SAFS) showed a significant effect. The tasks with small amplitudes evoked more freezing than the normal amplitudes, and the fast speed tasks evoked more freezing than the normal speed tasks. The ANOVA for the iTBS protocol showed the same results (Table S1). The ANOVA for the cTBS protocol showed only a significant effect for the factor time on FOUL of the least affected hand (Table S2). Post hoc analyses showed a decrease in FOUL duration (1.4 s pre-cTBS versus 1.2 s post-cTBS; *p* = 0.045) averaged over all tasks, but did not show a significant effect for one of the separate tasks.

Similar to the result for FOUL duration, the fixed factor task showed a significant effect on amplitude and frequency (Table 2, S1 and S2). In addition the ANOVA for the iTBS protocol showed an effect for the interaction of factors time and task on the amplitude of the most affected hand (Table S1). And although three tasks showed an increase in amplitude after the stimulation, post hoc analyses did not identify a significant effect on one of the separate tasks.

### Pegboard

The pegboard dexterity test did not show a difference in execution time between pre- and post-stimulation for both TBS protocols (cTBS and iTBS) in both the most and least affected hand.

### Corticospinal excitability

Both cTBS and iTBS did not have a significant effect on the corticospinal excitability over time measured over the M1 contralateral to the most affected side, when taking all three time points into consideration (pre, post 1, post 2) (factor time: *F*_2,32_ = 1.181; *p* = 0.320, factor stimulation: *F*_1,16_ = 0.518; *p* = n.s.). Neither was a significant effect measured for both cTBS (*p* = 0.820) and iTBS (*p* = 0.130), when only pre and post 1 were taken into consideration.

## Discussion

We tested the hypothesis that PD patients with FOG, who may have reduced cerebellar compensatory drive for motor function, would benefit from repetitive transcranial magnetic stimulation of the cerebellum. The main result is that both TBS protocols (facilitatory iTBS and inhibitory cTBS) did not significantly alter freezing duration in the upper limbs, nor during gait. However, an increase in overall gait speed when walking with small steps was found after TBS (decreased execution time), while gait speed during fast walking decreased after TBS. Additional analyses identified these changes to be primarily present after facilitatory iTBS.

We stimulated the cerebellum, because previous studies suggested that the role of the cerebellum in motor control of PD patients is compensatory [11, 12, 14–16]. Although our hypothesis about compensatory cerebellar activity preventing freezing in PD has not been confirmed, the changes in gait speed do suggest that improved gait performance is possible after cerebellar TBS.

### Gait effects and FOG

In PD patients with FOG an increased functional connectivity between the SMA on the one hand, and the cerebellum and the mesencephalic locomotor region on the other hand, was found during rest [9]. This emphasizes the importance of the cerebellum in this specific PD population. As an increase in functional connectivity was correlated with objective ratings of freezing, it was proposed that this reflects maladaptive compensation in FOG. However, as correlations do not reflect causality, an increase in functional connectivity could also indicate an increase in compensational strength of the network with increasing severity of FOG.

The importance of cerebellar activity in PD patients with FOG was confirmed as the gait protocol showed significant changes in the execution times, i.e. in gait speed. However, the hypothesized changes in FOG duration were not found. A possible reason for this result is the sensitivity to detect changes. In line with previous experiments, FOG proved difficult to elicit [33]. More repetitions in the gait protocol, especially the most FOG provocative tasks, could have increased the statistical power. In addition, the power of the study (and in particular the number of actually observed FOG events during the experiments) may have been too low to find changes in FOG.

Another reason could be that cerebellar TBS cannot sufficiently improve the complex neural circuitry that is involved specifically in the occurrence of FOG. For example, brainstem motor regions have also been associated with FOG [6, 44, 45]. Moreover, pedunculopontine nucleus (PPN) stimulation, a form of deep brain stimulation, successfully reduced the number of FOG episodes [46], albeit not consistently. Possibly, stimulation of specific regions is necessary and global cerebellar stimulation lacks such specificity.

### FOUL

We evaluated not only the effects of cerebellar TBS on freezing during gait, but also the effects on upper limb freezing. Similar to the results on FOG, no changes in FOUL duration were found after cerebellar TBS in the most affected hand, or in the pegboard performance (to test for hand bradykinesia). An effect of time was found for the least affected hand in the cTBS condition averaged over all tasks, but not for the tasks separately.

Previous fMRI studies of upper limb motion in PD patients without FOG have consistently shown increased activation in premotor-parietal and cerebellar regions. The increase in cerebellar regions was interpreted as a compensatory shift for the dysfunctional striato-supplementary motor loop [16, 47] and thought to influence the activity in the M1 through cerebellar-motor connections. The present results do not confirm such a compensatory role of the cerebellum in upper limb motor control.

### Corticospinal circuitry

The changes in gait speed were not accompanied by changes in MEP amplitude in the relaxed FDI of the most affected hand measured after cerebellar TBS. This lack of an effect on corticospinal excitability is in agreement with previous results in PD [48]. This suggests that cerebellar TBS does not affect the direct output from M1 of PD patients. An alteration of the cerebello-cortical connectivity after cerebellar rTMS has been found for PD patients [29, 30], but not always [48]. It could be that the increased gait speed when walking with small steps after TBS was accompanied by an alteration of cerebello-cortical connectivity, but not by an alteration of the motor cortex activity.

It is also possible that changes in gait speed are not accompanied by changes in MEP amplitude of hand muscles, but rather by changes in corticospinal excitability of leg muscles. In future studies it would be interesting to test the corticospinal excitability and cerebello-cortical connectivity of the lower limbs.

### Future perspectives

The results from this exploratory study provide more insight into the involvement of the cerebellum in gait mechanisms of PD patients with FOG. Our hypothesis about compensatory cerebellar activity preventing freezing in PD was not confirmed, but the results do provide multiple leads for future research.

Although the lateral cerebellum is involved in gait [49], the medial cerebellum could possibly be a better target location for future stimulation protocols. The medial cerebellum is important for balance and gait control [50, 51], and patients with medial cerebellar atrophy have gait and stance problems [49].

Another factor was our choice for unilateral instead of bilateral stimulation. Bilateral stimulation might be needed to compensate for freezing in both legs, although the dominant view is that reducing the asymmetry of gait parameters improves FOG [52]. Bilateral stimulation should include an asymmetry, wherein the least affected side is stimulated less than the most affected side [52].

We used a single session of cerebellar TBS instead of multiple sessions over a certain period. In a previous study also the effects of multiple sessions of cerebellar TBS have been evaluated [28]. Multiple sessions could possibly enhance the effects of the stimulation and maybe prolong them as well, which could thereby increase the effect on gait speed and possibly even cause an effect on FOG duration.

To identify the cerebellar and cerebral effects of the TBS that accompany the gait effects, future studies should combine stimulation sessions with fMRI or PET [53]. This would also help to establish how and to what extent activity in the cerebellum and the connected circuitries is affected by TBS.

A limitation of our study was the absence of a sham condition. We purposely made this choice to minimize the burden for patients, and only included two active interventions (cTBS and iTBS session), such that the contrast in excitatory effects and direction of the effect for both active conditions could serve as control for the opposite condition. Patients had to be tested ‘off’ medication to increase the possibility to observe FOG, which has a high impact on mobility [1, 2] on the days of testing. Consequently, placebo effects cannot fully be ruled out. However, if present, these placebo effects should be the same for all gait and stimulation conditions, as the patients were not aware of the nature of the stimulation, and because conditions were randomized. As TBS affected performance differently in different gait tasks, the results still suggest an involvement of the cerebellum in gait of PD patients with FOG. To test the hypothesized compensational mechanism of the cerebellum, future studies should include a sham condition.

Finally, stimulation of the cerebellum with TBS and other techniques will only become clinically relevant for future therapies when accompanied by a substantial decrease in freezing, and not only by changes in gait speed. Therefore, future studies in larger patient populations are needed with the aim of achieving an effect on freezing duration by increasing stimulation strength or by patient-specific targeting of the cerebellum. To compensate for the transient effect of rTMS, cerebellar transcranial direct current stimulation (tDCS) could be used instead [54]. An advantage of this technique is the possibility to stimulate the cerebellum during execution of the freezing evocative tasks.

## Electronic supplementary material

Below is the link to the electronic supplementary material.

Supplementary material 1 (DOCX 21 kb)

## Abbreviations

CBI: Cerebellar brain inhibition
cTBS: Continuous TBS
FAB: Frontal assessment battery
FDI: First dorsal interosseous
fMRI: Functional magnetic imaging
FOG: Freezing of gait
FOUL: Freezing of upper limbs
iTBS: Intermittent TBS
M1: Primary motor cortex
MDS-UPDRS: Movement Disorders Society—unified Parkinson’s disease rating scale
MEP: Motor evoked potential
MMSE: Mini mental state examination
N-FOGQ: New freezing of gait questionnaire
PD: Parkinson’s disease
PPN: Pedunculopontine nucleus
PSP: Progressive supranuclear palsy
rTMS: Repetitive TMS
SEM: Standard error of mean
SMA: Supplementary motor area
TBS: Theta burst stimulation
TMS: Transcranial magnetic stimulation
