# Supplementary material for: Cerebellar theta burst stimulation does not improve freezing of gait in patients with Parkinson’s disease
Source: J Neurol. 2017 Apr 5;264(5):963–72. doi: 10.1007/s00415-017-8479-y (PMC5413528; doi:10.1007/s00415-017-8479-y)
Supplement: Supplementary file 1 — Supplementary material 1 (DOCX 21 kb) [file 415_2017_8479_MOESM1_ESM.docx]

**Table S1.** Statistics gait task and upper limb task for iTBS.

|  | **Time** | **Task** | **Time x Task** |
| --- | --- | --- | --- |
| FOG duration  [combined] | [0.648; n.s.] | [0.934; n.s.] | [0.772; n.s.] |
| FOG duration  [separate] | [0.689; n.s.] | [0.950; n.s.] | [1.043; 0.398] |
| Mean execution time  [separate] | [**4.508; 0.049**] | **[3.779; 0.004]** | **[3.329; 0.009]** |
|  |  |  |  |
| FOUL duration [most] | [2.132; 0.166] | **[3.677; 0.019]** | [2.679; 0.060] |
| FOUL duration [least] | [0.530; n.s.] | [2.626; 0.063] | [0.274; n.s.] |
| Amplitude [most] | [0.350; n.s.] | **[44.601; 0.000]** | **[4.135; 0.012]** |
| Amplitude [least] | [0.067; n.s.] | **[85.682; 0.000]** | [1.443; 0.245] |
| Frequency [most] | [0.033; n.s.] | **[6.040; 0.002]** | [2.566; 0.068] |
| Frequency [least] | [0.200; n.s.] | **[13.084; 0.000]** | [0.371; n.s.] |

The factors are ‘*time’* (*pre* or *post*) and task. For gait the task includes (*normal, fast, small steps*, *small fast steps, turning clockwise or turning counter-clockwise*) and for upper limb (*NANS, NAFS, SANS* or *SAFS*). Factor task and interactions with factor task in separate FOG duration and mean execution time = [*F_5,16_; p*]. All other factors and interactions in FOG and mean execution time = [*F_1,16_; p*]. FOG duration was analyzed for all gait tasks [separately, 6 task conditions] and for turns and gait trajectory [combined, 2 task conditions] combined. Factor task and interactions with factor task for FOUL duration, amplitude and frequency = [*F_3,14_ ; p*]. All other factors and interactions for FOUL duration, amplitude and frequency = [*F_1,14_ ; p*]. Significant results are indicated in bold.

**Table S2.** Statistics gait task and upper limb task for cTBS.

|  | **Time** | **Task** | **Time x Task** |
| --- | --- | --- | --- |
| FOG duration  [combined] | [0.898; n.s.] | [0.843; n.s.] | [0.772; n.s.] |
| FOG duration  [separate] | [0.862; n.s.] | [1.251; n.s.] | [1.052; 0.394] |
| Mean execution time  [separate] | [0.153; n.s.] | **[21.470; 0.000]** | [0.840; n.s.] |
|  |  |  |  |
| FOUL duration [most] | [3.381; 0.087] | **[3.371; 0.027]** | [0.335; n.s.] |
| FOUL duration [least] | **[5.528; 0.033]** | [1.777; 0.166] | [1.502; 0.228] |
| Amplitude [most] | [0.072; n.s.] | **[33.951; 0.000]** | [0.534; n.s.] |
| Amplitude [least] | [0.054; n.s.] | **[37.189; 0.000]** | [0.512; n.s.] |
| Frequency [most] | [3.962; 0.068] | **[6.176; 0.001]** | [0.602; n.s.] |
| Frequency [least] | [0.859; n.s.] | **[11.389; 0.000]** | [0.893; n.s.] |

The factors are ‘*time’* (*pre* or *post*) and task. For gait the task includes (*normal, fast, small steps*, *small fast steps, turning clockwise or turning counter-clockwise*) and for upper limb (*NANS, NAFS, SANS* or *SAFS*). Factor task and interactions with factor task in separate FOG duration and mean execution time = [*F_5,16_; p*]. All other factors and interactions in FOG and mean execution time = [*F_1,16_; p*]. FOG duration was analyzed for all gait tasks [separately, 6 task conditions] and for turns and gait trajectory [combined, 2 task conditions] combined. Factor task and interactions with factor task for FOUL duration, amplitude and frequency = [*F_3,14_ ; p*]. All other factors and interactions for FOUL duration, amplitude and frequency = [*F_1,14_ ; p*]. Significant results are indicated in bold.
